# Small-Scale Fabrication of Biomimetic Structures for Periodontal Regeneration

**DOI:** 10.3389/fphys.2016.00006

**Published:** 2016-02-12

**Authors:** David W. Green, Jung-Seok Lee, Han-Sung Jung

**Affiliations:** ^1^Division in Anatomy and Developmental Biology, Department of Oral Biology, Oral Science Research Center, BK21 PLUS Project, Yonsei University College of DentistrySeoul, South Korea; ^2^Oral Biosciences, Faculty of Dentistry, The University of Hong KongHong Kong, Hong Kong; ^3^Department of Periodontology, Research Institute for Periodontal Regeneration, Yonsei University College of DentistrySeoul, South Korea

**Keywords:** periodontium, tissue engineering, modular biomaterials, 3D bioprinting, cell sheet engineering

## Abstract

The periodontium is the supporting tissues for the tooth organ and is vulnerable to destruction, arising from overpopulating pathogenic bacteria and spirochaetes. The presence of microbes together with host responses can destroy large parts of the periodontium sometimes leading tooth loss. Permanent tissue replacements are made possible with tissue engineering techniques. However, existing periodontal biomaterials cannot promote proper tissue architectures, necessary tissue volumes within the periodontal pocket and a “water-tight” barrier, to become clinically acceptable. New kinds of small-scale engineered biomaterials, with increasing biological complexity are needed to guide proper biomimetic regeneration of periodontal tissues. So the ability to make compound structures with small modules, filled with tissue components, is a promising design strategy for simulating the anatomical complexity of the periodotium attachment complexes along the tooth root and the abutment with the tooth collar. Anatomical structures such as, intima, adventitia, and special compartments such as the epithelial cell rests of Malassez or a stellate reticulum niche need to be engineered from the start of regeneration to produce proper periodontium replacement. It is our contention that the positioning of tissue components at the origin is also necessary to promote self-organizing cell–cell connections, cell–matrix connections. This leads to accelerated, synchronized and well-formed tissue architectures and anatomies. This strategy is a highly effective preparation for tackling periodontitis, periodontium tissue resorption, and to ultimately prevent tooth loss. Furthermore, such biomimetic tissue replacements will tackle problems associated with dental implant support and perimimplantitis.

## Introduction

The periodontium tissue complex is adapted to fixing and supporting the tooth into the mandibular and maxillary bone sockets and preserving its structure under extreme masticatory forces. Unfortunately, there are currently no permanent cures for chronic and advanced periodontal tissue degeneration. This situation has spurred efforts to grow new replacement patient-matched periodontal tissue for transplantation into the periodontal pocket. Arguably, this represents the only clear permanent solution for periodontal tissue degeneration.

To gain acceptance and to be relevant in the clinic, prospective engineered tissue replacements must display higher levels of tissue biomimicry, to promote coherent host integration. Biomimetic tissue systems can be engineered to program tissue morphogenesis and produce good clinical outcomes. How should tissue engineers design and produce structures that enable embedded tissue components to self-organize and spontaneously grow into proper tissues? Modular design of biomaterials may be a good starting point.

Modular biomaterials are a useful way of building tissue complexity in the laboratory. They are composites of limitless structural units. These units can have any type of size, composition, morphology, and topology. They can be fused together in any sort of position to make intricate patterns. When built-up in scale they can form hierarchies. The combination of structure and composition enables them to perform the numerous roles and behaviors of native tissue development and regeneration. This level of organization is not possible with conventional cell sheet engineering. Infact, tissue biology is highly modular itself. There are many delineations, partitionings, dissociations, associations, and complex organizations made between tissue components (cells, proteins, and matrices). By engineering in a modular fashion with assorted functional tissue units is to emulate native tissue organization. Assembling tissues in this manner will accelerate correct mimetic tissue morphogenesis and near normal tissue functioning. Once structure and function are synergized then tissues can remain permanent.

## Engineering criteria for periodontal regeneration

**Figure 1:**
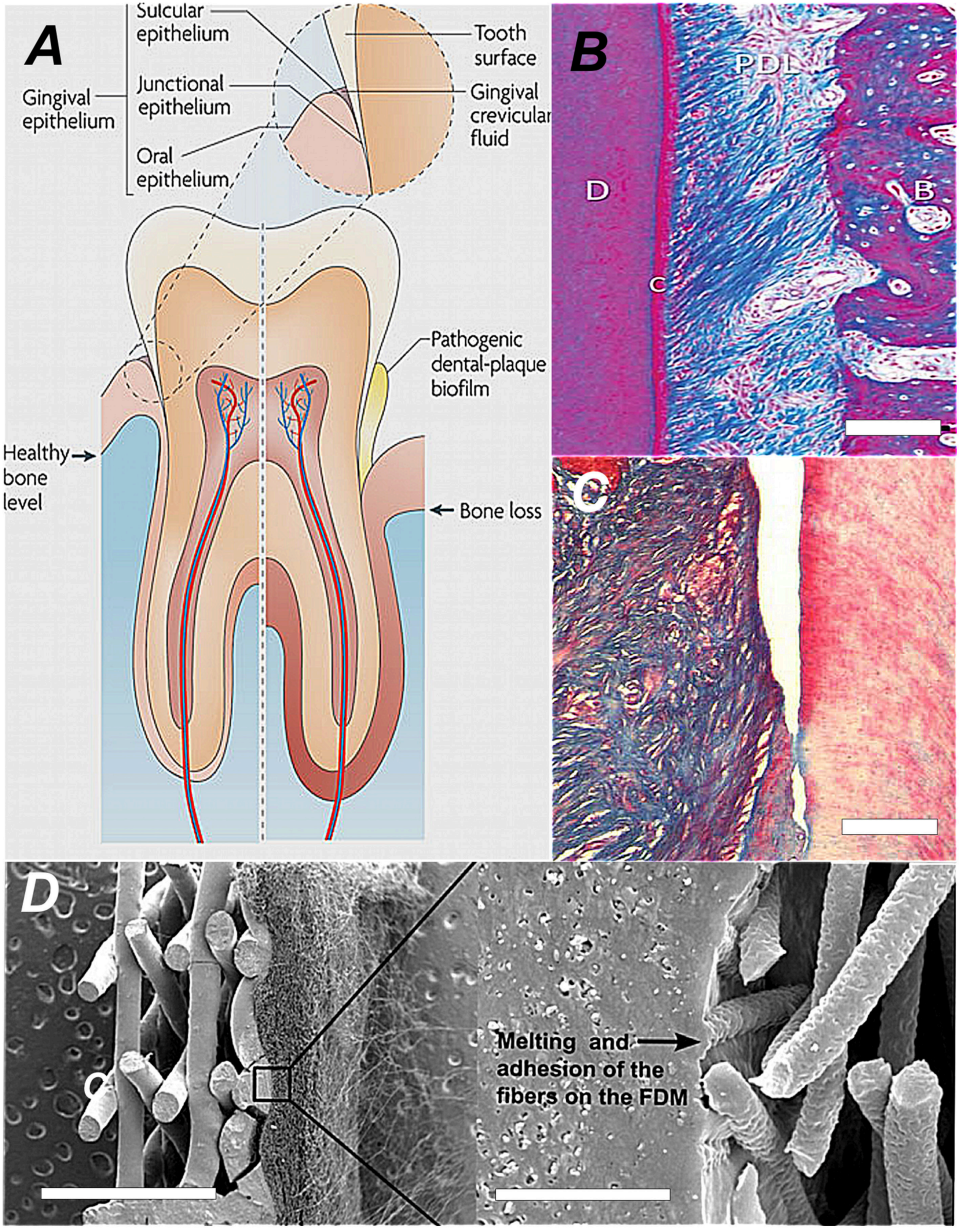
**The comparative anatomy and histoarchitecture of healthy and diseased periodontal tissue and a typical biomaterial replacement for lost union structures**. **(A)** A diagram showing the difference in the tooth supporting structures between normal healthy periodontium and diseased periodontium. In particular, the “periodontal connective tissue” and bone are destroyed by growth of a dental plaque biofilm that attaches and develops first at the sulcus, progressing along the tooth root and then creating a deepening pocket (Darveau, 2010; Reproduced with kind permission from Macmillan). **(B)** The healthy periodontal ligament at the centre of the periodontium complex shown in polarized light at high magnification. In this view we can see the densified collagen fiber bundles arranged in very high order corresponding to strong birefringence. Along the tooth root the collagen fiber mass is anchored and suspended between the cementum (C)/dentine and alveolar bone (AB). This image depicts the histoarchitecture of the periodontium. It shows the complex nature of the tissue architecture. The periodontal 93hinge94 ligament tissue is filled with cell assemblies and blood vessels servicing its role in continual cycles of readjustment, repair and regeneration. (Reproduced with kind permission from John Wiley and Sons A/S; Bosshardt and Sculean, 2009). **(C)** Close-up histosection of an advanced gingival mucosa lesion at the tooth surface caused by the infiltration of periodontitis causing bacteria. The periodontal ligament has clearly separated and peeled away from the tooth surface (dentine) on the right. Cementum is lost and the junctional epithelium disappears from the tooth surface as the gingival pocket opens-up. Tissue engineering approaches involve infilling the space and regenerating a new periodontium in between pulling a whole new union “cable” structure of collagen fibers (Type I) from a *de novo* periodontal ligament into reconstituted *de novo* alveolar bone and Sharpeys collagen fiber tips from the periodontal ligament embedded into the cementum at the tooth surface (Type III; Hasegawa et al., 2005; Reproduced with kind permission from Mary Anne Liebert). **(D)** One conventional biomaterial led strategy is simpy to combine thin layers of material with tissue-specific architectures that mimic periodontal ligament and the cementum layer. Each structure provides contact guidance for the proper organization of cementum cells and the alignment of the peridontal ligament fibers. The seeded cells collectively organize themselves into a coherent functional unit. The SEM image shows an engineered bi-layered polymer structure that provides the appropriate physical framework for the periodontal ligament cells and the cementum. The PDL housing consists of an electrospun structure with fibrous mesh architecture, to align and guide the extension of PDL fibers. This meshwork was melted onto the porous solid layer with a technique called fused deposition modeling (FDM; Obregon et al., 2015; Reproduced with kind permission from Elsevier).

Conventional periodontal clinical therapy focuses on bacteria removal (plaque control), pocket depth reduction by surgical reattachment, reattachment to the root surfaces, forming a hermetic epithelial barrier, and tackling inflammation (Figures 1A,B). However, the grand prize for periodontal therapy is the total replacement of missing and degenerate tissue with healthy functional tissue originating from patient cells. This is only achieved by creating proper conditions for *de novo* tissue formation engineered in the laboratory or inside the periodontal pocket (Figure 1C). There are two dimensions to periodontium tissue regeneration. There is the attachment complex along the root of the tooth and the attachment complex that forms the epithelial seal against the neck of the tooth masticatory surface (Figure 1A; left side of tooth diagram). These union structures are partitioned into numerous anatomical compartments, (some well-delineated and some diffuse) each typified with its own specialized functional properties, ultrastructure, size and morphology.

To ensure fast, effective, and accurate biomimetic translation from concept to clinic, these compartments must be stacked together as layers (between 5 and 100 μm) in a specific functional sequence and fixed together with interconnecting embedded union structures made from regionally specialized collagen fibers (Figure 1B). Another compartment of significance to driving regeneration of cementum is the epithelial cell rests of Malassez. Modular design can incorporate this cellular assembly and other stem cell niche compartments as adjuncts. Ultimately, this must be a synchronized production from collective cellular programming and self-organization biological processes. These are the prime drivers, for producing final functional complexity. The proper positioning of compartments establishes a set of functional pre-arrangements of cells and matrix components that spontaneously and inevitably give rise to correct tissue ultrastructures, morphologies, and anatomies.

Strategies being developed for periodontal regeneration are embracing numerous tissue engineering techniques to recreate a living replacement that can spontaneously graft into the walls of periodontal pocket and so provide fresh, permanent replacement to re-support the compromised tooth organ. To generate high volumes of good quality biomimetic tissue the physical environment for growth and regeneration needs to emulate the physical (e.g., mechanical, patterning) and topological complexity of normal periodontium structures. Modular biomaterials can be a good beginning for representing this organizational complexity and an approach for translating biocomplexity into tissue engineered biosystems. For instance, modular biomaterials will be capable of mapping networks and relationships between cells and components that promote self-regeneration processes. In this short article, we focus exclusively on modular biomaterials to engineer the periodontium and speculate on how this can improve tissue engineered outcomes.

## Frameworks to support periodontal tissue regeneration

The engineering of periodontal tissue sets is at a crossroads of approaches: biomaterial-free tissue morphogenesis, tissue morphogenesis with biomaterial engagement of endogenous regeneration, and fully engaged biomaterials seeded with cells. Biomaterials with regeneration potential have nominally provided transient stop-gaps and bridges across the periodontal pocket.

An important contribution to the existing panel of engineered periodontal replacements has been essentially devoid of biomaterials involving cells assembled into sheets (Asakawa et al., 2010). Cell sheet engineering has been widely used to construct tissues with translation into stratified tissue types and simple anatomical cellular organizations such as, the skin, myocardium, corneal, and mucosal epithelium as well as, PDL, cementum, and the alveolar bone lining (Iwata et al., 2009). It is a relatively low-tech and effective fabrication method. There is a capacity with this technique to grow coherent, self-supporting monolayers of tissue-specific cells and then deploy them as stratified constructs into small tissue defects. Cell sheets and multiple sheets have also been used to encase implantable devices (not exceeding 3–6 layers or 80–100 μm). Cell sheets are reminiscent of the organization of periodontal tissues and have been used in reconstructions. There is an attempt to replicate the different layers using more rationally designed material frameworks to restore different tissue morphologies. In one Periodontal related example, electrospinning (to create a fibrous network) and fused deposition modeling (FDM; to create a small porous solid membrane) recreated the PDL and mineralized layer of bone or cementum respectively (Obregon et al., 2015; Figure 1D).

**Figure 2:**
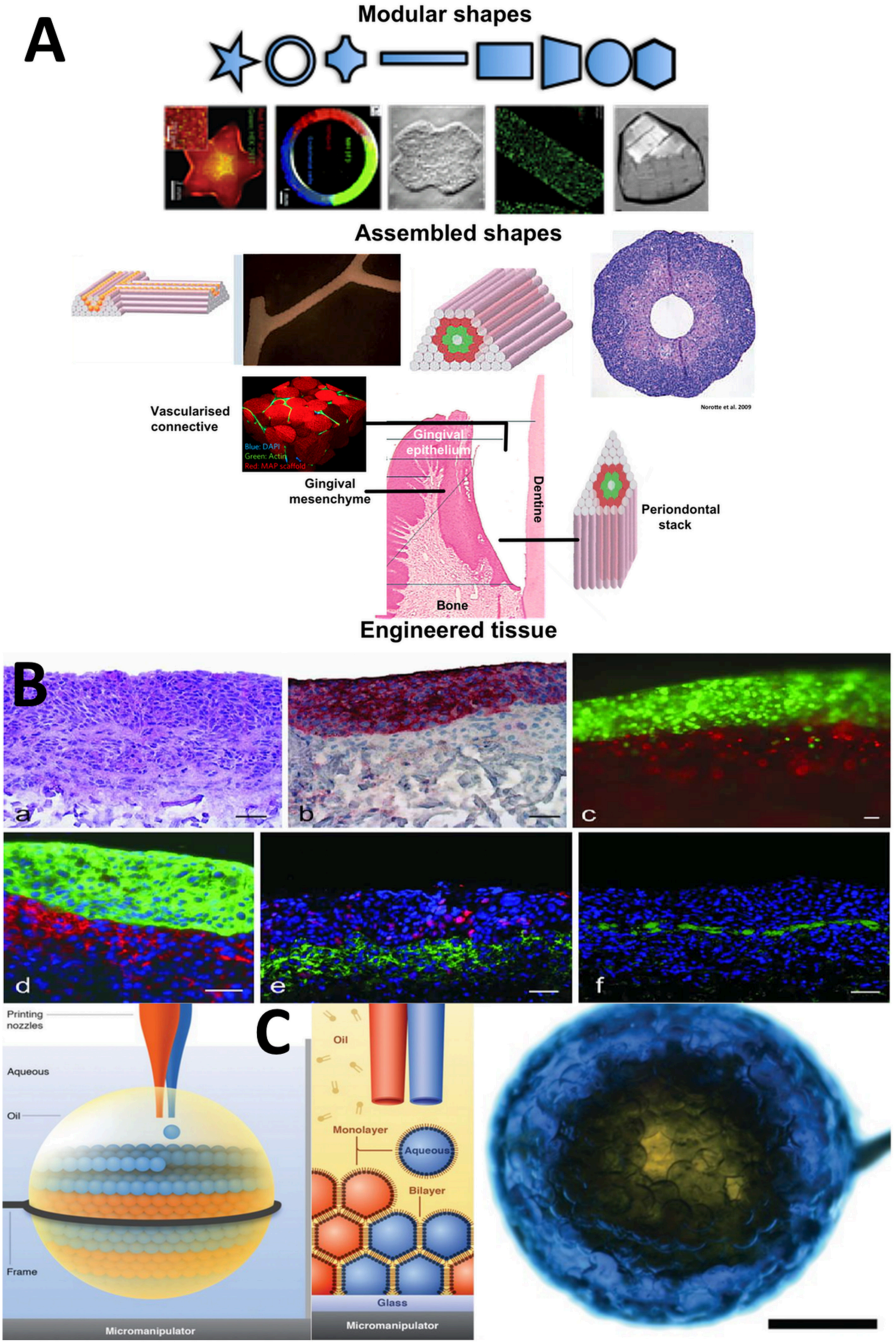
**Selected Microfabricated modular biomaterials by laser printing, 3D microdroplet printing and microdroplet fluidics. (A)** (a) A diagram showing the basic concept for modular biomaterials design. Particular shaped blocks of material (mainly hydrogels) are created by one of the micro fabrication techniques listed. The assembly of those building blocks can be directed or allowed to randomly self-assemble. It is then possible to generate large blocks of material using combinations of the small modules. A range of different module shapes, that have been fabricated, is shown in (b). In (c) we present a diagram showing the different module structures and organization that could be used to represent the various periodontium tissues structurally and compositionally in a very basic representation to mimic the structural environments necessary for gingival epithelium development, subepithelial connective tissue formation and the stacked layers of cementum, ligament and the alveolar bone lining (McGuigan and Sefton, 2006; McGuigan et al., 2006, 2008; Nichol and Khademhosseini, 2009; Chamberlain et al., 2010; Reproduced with kind permission from PLoS, MacMillan Publishing, MYJoVE Corporation, American Association for the Advancement of Science, Royal Society of Chemistry). **(B)** (a) Showing the arrangement of printed cells in a distribution reminiscent of skin tissue; (b) Red staining to highlight the distinct keratinocyte cell region; (c) Fluorescently labeled red fibroblasts and green keratinocytes printed using the laser technique into tight and clear layers; (d) Phenotype related fluorescence tagging of fibroblasts (red) and keratinocytes for cytokeratin 14 expression. Note the densification of cells in this layer; (e) Fluorescent labeling of the printed cellular construct for proliferation using red Ki-67 and in (f) for presence of laminin basement membrane protein in green, which forms a distinct supporting layer (all scale bars = 50 microns). “Laser-Assisted BioPrinting” was used to manufacture a layered skin reproduction, which can easily be translated to other multi-layered tissues such as, the periodontium. In this microfabrication technique, a solution containing fibroblasts and keratinocytes were ejected from the solution as a continuous jet of droplets using laser force field. The droplets are collected into compound structures, onto a solid receiver substrate, where the solution is able to gel. New layers are stacked-up on the preceding layer using the same procedure. Different cell types of the skin were infused into the different layers representing the native cellular composition of human skin. This fast, automated type of layering procedure can be easily tailored to recreate the laminate “sandwich” of tissues in the periodontium (Koch et al., 2012; Reproduced with kind Permission from Wiley) **(C)** An ordered, aggregated array of droplets that can mimic certain forms of simple tissue structures. Three-dimensional printing is used to arrange assorted aqueous droplets (modules) into highly organized globular shaped microstructures. The water droplets are printed into “lipid-containing oil droplets” and coated in a lipid bi-layer. As well they are supported inside the macrodroplet. The lipid provides a soft substrate in which membrane proteins can be inserted. This provides channels between individual droplet-based mdules. The whole process is precisely controlled by the printer program settings so that diverse arrangements of architectures are conceived via computer-aided designs (Villar et al., 2013) (Reproduced with Kind permission of the American Association for the Advancement of Science).

Modular biomaterials are tissue engineering structures divided into a network of small segments containing tissue components tailored toward synergistic and synchronized function between neighboring segments (Zorlutuna et al., 2013). The cell-containing building blocks are arranged so as to mimic the organization of native tissue. Traditional cell-seeded frameworks are a scramble of tissue components. The modular approach is a way of ordering tissue components according to native anatomical organization, which is always diverse and complex (Figure 2A). These design principles when applied to the periodontium then translate into semi-permeable layers, with physical guides to direct the fiber alignments across different regions, for the PDL and cementum. They translate into spheres and rods to form a template for epithelialization and barrier formation and spherical outgrowths representing regional niches and crypts for epithelial cell rests of Malassez ERM's and stem cell phenotypes. Modular design facilitates the accumulation of cellular assemblies and other stem cell niche compartments. Ultimately, this must be a synchronized production from collective cellular programming and self-organization biological processes. These are the prime drivers, for producing the final functional complexity. The proper positioning of compartments establishes a set of functional pre-arrangements of cells and matrix components that spontaneously and inevitably give rise to correct tissue ultrastructures, morphologies, and anatomies.

Now there are some notable module structures designed to house assorted cell populations and phenotypes. There are various methods to assemble them in rational networks related to selected tissue architecture (Khademhosseini et al., 2006; Gauvin and Khademhosseini, 2011; Hansen et al., 2013; Kolesky et al., 2014). Bioprinting in three-dimensions (programmable positioning of building blocks) combined with microfluidic technology (programmable size and shape generator of droplets for building blocks) could propel the manufacture of more highly intricate and complex morphologies and the deep internal structures and architectures that more accurately replicate the exact anatomical organization of many complex tissue and including periodontal tissues with its multi-planar differences in composition and architecture (Erdman et al., 2014). A standardized microengineering method of producing regular modules is with polymer microdroplets. Changes in droplet size (in nanoliter and microliter volumes) (Gruene et al., 2011), composition and deposition leads to single modules and module networks with biologically acceptable complexity (Durmus et al., 2013). Three-dimensional bioprinting and aqueous droplet microfluidics and nanofluidics are the highest precision tools in generating and depositing mixtures of aqueous biomaterials into precisely defined shapes, structures (including internalized structures) and assemblies of droplets and beads with well-delineated architectures at micron scales and nanoscales with picoliter droplets (Erdman et al., 2014; Murphy and Atala, 2014). Contained within the droplets are mixtures of cells, tissue components, and biological molecules. Blood vessel networks are critical to the development of new tissues. Microprinting technology has been used to generate branched networks of tubules mimicking natural blood vessels (Kucukgul et al., 2015; Wang et al., 2015). Repeated gelling of hydrogels, one on top of another, placed within geometrically patterned templates was used to generate hexagonal shaped hepatic structures (Liu Tsang et al., 2007). The arrangement of modules into deliberate geometries and shapes is controlled by deposition of aqueous hydrophilic droplets, onto a hydrophobic oil receiver solution (Nichol and Khademhosseini, 2009; Du et al., 2011). Chemical reactions between hydrophilic and hydrophobic components are a driving force for organization and patterning of objects into the directed geometries. This is almost 100% controlled. As with any fabrication tool the sizes of the individual modules and the networks are limited. Modular-based architectures also provide temporal and spatial patterning capabilities for vital processes involved in development and regeneration. There are instances where this has been delivered for the controlled and regulated elution of growth factors using core-shell arrangements and bead-in-bead arrangements (Babister et al., 2008; Perez et al., 2014; Perez and Kim, 2015).

Modular biomaterials represent a potential solution to provide temporary structures and architectures for the precise positioning of cells and associated factors (to generate proper tissues) in 3D space that accurately match normal tissue and lead to functional associations toward proper regeneration. A one-to-one biomimetic matching between the smallest units present in natural tissue and the replicated modules is not necessary for function. Function is designed to emerge from the interplay between the well-placed tissue components inside the combined modules. The fusion of tissue modules into clusters and networks establishes a provisional tissue mimicking anatomical structure. These arrangements also facilitate the directional release of factors designed to stimulate different types of pre-existing endogenous tissues to grow and develop into the modular replacement.

## Tissue biomimicry with modular biomaterials

Tissues are essentially divided into compartments bounded by cell linings (intima, adventitia), membranes (vesicles and pouches), and boundaries made from structural biomaterials. The intricate complexity of form and architecture are strongly and directly associated with function. There is a growing interest in designing biomaterial devices and products that reflect this compartmentalization arrangement, which facilitates, partitioning, gradation, and division of tissue entities (Nichol and Khademhosseini, 2009; Figures 2A,B). Crucially, the building of compartments relies on chemical self-organization processes rather than biologically driven self-assembly and self-organization processes toward tissue complexity (Jakab et al., 2010; Athanasiou et al., 2013). These can also be directed in specified ways by manipulating the steps in synthesis. The most commonly used and versatile semi-autonomous system is hydrogel in oil at interfaces and with droplets, which enables well-delineated shapes to be generated (Gauvin and Khademhosseini, 2011). Another chemically driven system employs capillary forces to force microgel units into packing arrangements that can support themselves in tight ball and rod-like structures, for example (Fernandez and Khademhosseini, 2010). Using DNA links attached to their external surfaces, building blocks of microgel are instilled with a program for self-assembly or piecing together in precise ways coded for by the complementarity of the DNA molecules (Qi et al., 2013).

Modular biomaterial strategies have tended to employ small, single blocks of material in a thus-far limited selection of shapes (typically spheres and rods, the products of chemical laws used in their formation in aqueous solutions; Figures 2A,C) with variable dimensions. These small building blocks are constructed into high order architectures by three defined assembly routes: random assembly, which is governed by the chemical and physical properties of the chemical environment; stacking together layers of material; or by directed assembly in which the modules are purposefully pieced together by the action of external forces or by molecular “lock and key” decorations at the surfaces of modules (Nichol and Khademhosseini, 2009; Liu et al., 2010). However, the scale is limited to an individual function and encapsulating modules inside a bulk material. An early example was the construction of collagen microrods encased in endothelial cells (McGuigan et al., 2008). Microfluidic engineering is a powerful method for compartmentalizing many types of tissue-specific cells and microtissues for the regeneration of various tissues, production of organoids and synthesis of vascular-branched networks (Lu et al., 2015). Modular microtissues supported inside collagen-fibrin beads, up to 300 μm in diameter, grew internal vascular networks (vascularization). Embedded inside a gel, the networks extended beyond the original beads into vessel structures that formed networks, mimicking angiogenesis, after 14 days (Peterson et al., 2014). Modular biomaterials have gained popularity in drug delivery, targeting therapeutics and for immunotherapy. The increasingly complex shaping of modules is made possible by using complex fluids such as with mixtures of polyelectrolytes and surfactants (Lapitsky et al., 2008). Compartments built with biomaterials are referred to as modules. The ability to create modules small enough to mirror those in nature has profoundly increased due to advances in microfabrication strategies (Zorlutuna et al., 2012). Modules of different shapes and sizes can be printed with inkjets or molded using lithography (Figures 2B,C).

## Transferring modular biomaterials into the dental clinic

We have described an idea about how to compartmentalize and arrange cells and their supporting components into arrangements that imitate the basic organization of the tissue units. We have provided examples of the first steps, where this has been used to mimic hepatic tissue, and structures with hexagonal packing such as, retinal pigment epithelium, corneal endothelium, and kidney papillary ducts. Layer-by-layer reconstructions with regional variations can be recreated with high aspect ratio compartments filled with nested modules, compounded too with modules representing folds and pouches containing regenerative progenitors (Nanci and Bosshardt, 2006). An important biomimetic property for the modules, housing specialized tissue, and cell specific microenvironments, is to have interfaces that permit physical and molecular exchanges with apposing tissue compartments so that growth factor receptors are bound and cell-to-cell and cell-matrix junction proteins connections are established. These are needed for self-organization (via cell fusion, differential cell adhesions and contact minimizations) and tissue morphogenesis. The positioning arrangements of the individual periodontium tissue types (and the anatomical sub-types) must be synchronized and coordinated to create the tight biological and mechanical interlocking of bone with ligamentous tissue, with cementum and with dentine along the root and gingiva epithelium, mesenchyme and epithelium at the enamel/dentine junction of the tooth. The positioning, integration, connectivity, and coordination of the anatomical and functional units of multiple tissues can be achieved using one of the various microfabrication technologies ranging between microfluidics to 3D printing techniques (Figure 2). Regional variations in tissue dimensionality and tissue component density, distributions, types of co-mixtures, and gradations can be engineered with modular approaches.

To improve the interplay between the components of distinct units, we anticipate constructing compartment boundaries that replicate the behavior of basement membranes and cell linings in which the cross-boundary throughput of biomolecules can be autonomously regulated by including membrane voltage-gated ion channels (out of 300), water channels (aquaporins), and cell integrins receptors (attachment and signal transduction), contact junctions, and cell-and matrix specific ligands. The design must also facilitate cell-to-cell connections and cross-migrations between compartments and networks of modules.

We have described numerous ways of building the individual microscale modules and microtissue units into larger macrostructures that can bridge the gap of the periodontal pocket. “Layer-by-layer” building designs suit the specific sandwich design (tooth–cementum–PDL–bone) of the natural human periodontium. Cell sheet engineering has created impressive reconstructions of the periodontal ligament, but the low initial mechanical properties, low blood supply through an interpenetrating vasculature and regulation of growth *in situ* are unresolved problems of cell sheets (Matsuda et al., 2007; Sawa and Miyagawa, 2013).

To achieve clinical relevance and acceptability, the modularization technique must incorporate specific tissue microenvironments that trigger and drive the development of each tissue type along its pre-programmed pathways and cascade. The individual tailoring of module microenvironments, so significant in delineating cell phenotype, proliferation, apoptosis and future fates, has already been demonstrated, as has the packing together of modules holding different environmental qualities (Guillame-Gentil et al., 2010). The partitioning and re-aggregation of such units will facilitate the programming of dynamic changes, in line with transitional phases in development and morphogenesis, as materials can be engineered with environmental responsiveness to external cues in regeneration. In addition, the modular approach allows for the integration of spaces for vasculature templating.

We have introduced a concept to biomaterials design that is not mainstream and has not been thoroughly explored as strategy for biomimetic translation of tissue organization from nature to the laboratory. The hardware is available to construct appropriate modules with sizes, shapes and attachments, which bring together tissue components and mimic tissue anatomical organization. One of the greatest unment challenges for modular tissue engineering is simulating structures for blood vessel networks without which the engineered tissue is unusable. Models for biomimicry include adult tissues as well as embryonic tooth development. Maps on how to arrange modules and their tissue components in biomimetic fashion will need to be drawn, trialed and tested for the best clinical outcomes in treating periodontitis and supporting failing and infected dental implants.

## Funding

This work was supported by the National Research Foundation of Korea (NRF) grant funded by the Korea government (MSIP) (No. 2014R1A2A1A11050764). This work was supported by the Korean Federation of Science and Technology Societies (KOFST) grant funded by the Korean government (MSIP: Ministry of Science, ICT and Future Planning).

### Conflict of interest statement

The authors declare that the research was conducted in the absence of any commercial or financial relationships that could be construed as a potential conflict of interest.

## References

[B1] Asakawa N. Shimizu T. Tsuda Y. Sekiya S. Sasagawa T. Yamato M.. (2010). Pre-vascularization of *in vitro* three-dimensional tissues created by cell sheet engineering. Biomaterials 14, 3903–3909. 10.1016/j.biomaterials.2010.01.105 20170957

[B2] Athanasiou K. A. Eswaramoorthy R. Hadidi P. Hu J. C. (2013). Self-organization and the self-assembling process in tissue engineering. Annu. Rev. Biomed. Eng. 15, 115–136. 10.1146/annurev-bioeng-071812-152423 23701238 PMC4420200

[B3] Babister J. C. Tare R. S. Green D. W. Inglis S. Mann S. Oreffo R. O. (2008). Genetic manipulation of human mesenchymal progenitors to promote chondrogenesis using “bead-in-bead” polysaccharide capsules. Biomaterials 29, 58–65. 10.1016/j.biomaterials.2007.09.006 17897711

[B4] Bosshardt D. D. Sculean A. (2009). Does periodontal tissue regeneration really work? Periodontol 2000 51, 208–219. 10.1111/j.1600-0757.2009.00317.x 19878476

[B5] Chamberlain M. D. Butler M. J. Ciucurel E. C. Fitzpatrick L. E. Khan O. F. Leung B. M.. (2010). Fabrication of micro-tissues using modules of collagen gel containing cells. J. Vis. Exp. 46:2177. 10.3791/2177 21178971 PMC3278332

[B6] Darveau R. P. (2010). Periodontitis: a polymicrobial disruption of host homeostasis. Nat. Rev. Microbiol. 7, 481–490. 10.1038/nrmicro2337 20514045

[B7] Du Y. Ghodousi M. Qi H. Haas N. Xiao W. Khademhosseini A. (2011). Sequential assembly of cell-laden hydrogel constructs to engineer vascular-like microchannels. Biotechnol. Bioeng. 7, 1693–1703. 10.1002/bit.23102 21337336 PMC3098307

[B8] Durmus N. G. Tasoglu S. Demirci U. (2013). Bioprinting: functional droplet networks. Nat. Mater. 6, 478–479. 10.1038/nmat3665 23695742

[B9] Erdman N. Schmidt L. Qin W. Yang X. Lin Y. DeSilva M. N.. (2014). Microfluidics-based laser cell-micropatterning system. Biofabrication 3:035025. 10.1088/1758-5082/6/3/035025 25190714 PMC4354940

[B10] Fernandez J. G. Khademhosseini A. (2010). Micro-masonry: construction of 3D structures by microscale self-assembly. Adv. Mater. Weinheim. 22, 2538–2541. 10.1002/adma.200903893 20440697 PMC2957829

[B11] Gauvin R. Khademhosseini A. (2011). Microscale technologies and modular approaches for tissue engineering: moving toward the fabrication of complex functional structures. ACS Nano. 5, 4258–4264. 10.1021/nn201826d 21627163 PMC3132595

[B12] Gruene M. Unger C. Koch L. Deiwick A. Chichkov B. (2011). Dispensing pico to nanolitre of a natural hydrogel by laser-assisted bioprinting. Biomed. Eng. 10:19. 10.1186/1475-925X-10-19 21385332 PMC3058070

[B13] Guillame-Gentil O. Semenov O. Roca A. S. Groth T. Zahn R. Vörös J.. (2010). Engineering the extracellular environment: strategies for building 2D and 3D cellular structures. Adv. Mater. Weinheim. 22, 5443–5462. 10.1002/adma.201001747 20842659

[B14] Hansen C. J. Aksena R. Kolesky D. B. Vericella J. J. Kranz S. J. Muldowney G. P.. (2013). High-throughput printing via microvascular multinozzle arrays. Adv. Mater. Weinheim. 25, 96–102. 10.1002/adma.201203321 23109104

[B15] Hasegawa M. Yamato M. Kikuchi A. Okano T. Ishikawa I. (2005). Human periodontal ligament cell sheets can regenerate periodontal ligament tissue in an athymic rat model. Tissue Eng. 11, 469–478. 10.1089/ten.2005.11.469 15869425

[B16] Iwata T. Yamato M. Tsuchioka H. Takagi R. Mukobata S. Washio K.. (2009). Periodontal regeneration with multi-layered periodontal ligament-derived cell sheets in a canine model. Biomaterials 14, 2716–2723. 10.1016/j.biomaterials.2009.01.032 19201461

[B17] Jakab K. Norotte C. Marga F. Murphy K. Vunjak-Novakovic G. Forgacs G. (2010). Tissue engineering by self-assembly and bio-printing of living cells. Biofabrication 2:022001. 10.1088/1758-5082/2/2/022001 20811127 PMC3635954

[B18] Khademhosseini A. Eng G. Yeh J. Fukuda J. Blumling J. III Langer R.. (2006). Micromolding of photocrosslinkable hyaluronic acid for cell encapsulation and entrapment. J. Biomed. Mater. Res. A. 79, 522–532. 10.1002/jbm.a.30821 16788972

[B19] Koch L. Deiwick A. Schlie S. Michael S. Gruene M. Coger V.. (2012). Skin tissue generation by laser cell printing. Biotechnol. Bioeng. 7, 1855–1863. 10.1002/bit.24455 22328297

[B20] Kolesky D. B. Truby R. L. Gladman A. S. Busbee T. A. Homan K. A. Lewis J. A. (2014). 3D bioprinting of vascularized, heterogeneous cell-laden tissue constructs. Adv. Mater. Weinheim. 26, 3124–3130. 10.1002/adma.201305506 24550124

[B21] Kucukgul C. Ozler S. B. Inci I. Karakas E. Irmak S. Gozuacik D.. (2015). 3D bioprinting of biomimetic aortic vascular constructs with self-supporting cells. Biotechnol. Bioeng. 112, 811–821. 10.1002/bit.25493 25384685

[B22] Lapitsky Y. Zahir T. Shoichet M. S. (2008). Modular biodegradable biomaterials from surfactant and polyelectrolyte mixtures. Biomacromolecules 1, 166–174. 10.1021/bm7009416 18088095

[B23] Liu B. Liu Y. Lewis A. K. Shen W. (2010). Modularly assembled porous cell-laden hydrogels. Biomaterials 31, 4918–4920. 10.1016/j.biomaterials.2010.02.069 20338634

[B24] Liu Tsang V. Chen A. A. Cho L. M. Jadin K. D. Sah R. L. DeLong S.. (2007). Fabrication of 3D hepatic tissues by additive photopatterning of cellular hydrogels. FASEB J. 21, 790–801. 10.1096/fj.06-7117com 17197384

[B25] Lu Y.-C. Song W. An D. Kim B. J. Scwartz R. Wu M. (2015). Designing compartmentalized hydrogel microparticles for cell encapsulation and scalable 3D cell culture. J. Mater. Chem. B. 3, 353–360. 10.1039/C4TB01735H 32262039

[B26] Matsuda N. Shimizu T. Yamato M. Okano T. (2007). Tissue Engineering Based on Cell Sheet Technology. Adv. Mater. Weinheim. 19, 3089–3099. 10.1002/adma.200701978

[B27] McGuigan A. P. Bruzewicz D. A. Glavan A. Butte M. J. Whitesides G. M. (2008). Cell encapsulation in sub-mm sized gel modules using replica molding. PLoS ONE 3:e2258. 10.1371/journal.pone.0002258 18493609 PMC2376064

[B28] McGuigan A. P. Leung B. Sefton M. V. (2006). Fabrication of cell-containing gel modules to assemble modular tissue-engineered constructs [corrected]. Nat Protoc. 1:2963–2969. 10.1038/nprot.2006.443 17406556

[B29] McGuigan A. P. Sefton M. V. (2006). Vascularized organoid engineered by modular assembly enables blood perfusion. Proc. Natl. Acad. Sci. U.S.A. 103, 11461–11466. 10.1073/pnas.0602740103 16864785 PMC1544192

[B30] Murphy S. V. Atala A. (2014). 3D bioprinting of tissues and organs. Nat. Biotechnol. 32, 773–785. 10.1038/nbt.2958 25093879

[B31] Nanci A. Bosshardt D. D. (2006). Structure of periodontal tissues in health and disease. Periodontology 2000 40, 11–28. 10.1111/j.1600-0757.2005.00141.x 16398683

[B32] Nichol J. W. Khademhosseini A. (2009). Modular tissue engineering: engineering biological tissues from the bottom up. Soft Matter. 5, 1312–1319. 10.1039/b814285h 20179781 PMC2826124

[B33] Obregon F. Vaquette C. Ivanovski S. Hutmacher D. W. Bertassoni L. E. (2015). Three-dimensional bioprinting for regenerative dentistry and craniofacial tissue engineering. J. Dent. Res. 94, 143S–152S. 10.1177/0022034515588885 26124216

[B34] Perez R. A. Kim H. W. (2015). Core-shell designed scaffolds for drug delivery and tissue engineering. Acta Biomater. 21, 2–19. 10.1016/j.actbio.2015.03.013 25792279

[B35] Perez R. A. Kim M. Kim T. H. Kim J. H. Lee J. H. Park J. H.. (2014). Utilizing core-shell fibrous collagen-alginate hydrogel cell delivery system for bone tissue engineering. Tissue Eng. A 20, 103–114. 10.1089/ten.tea.2013.0198 23924353 PMC3875185

[B36] Peterson A. W. Caldwell D. J. Rioja A. Y. Rao R. R. Putnam A. J. Stegemann J. P. (2014). Vasculogenesis and angiogenesis in modular collagen-fibrin microtissues. Biomater. Sci. 2, 1497–1508. 10.1039/C4BM00141A 25177487 PMC4145346

[B37] Qi H. Ghodousi M. Du Y. Grun C. Bae H. Yin P.. (2013). DNA directed self-assembly of shape-controlled hydrogels. Nat. commun. 4:2275. 10.1038/ncomms3275 24013352 PMC3768014

[B38] Sawa Y. Miyagawa S. (2013). Present and future perspectives on cell sheet-based myocardial regeneration therapy. BioMed. Res. Int. 2013:ID583912. 10.1155/2013/583912 24369013 PMC3867859

[B39] Villar G. Graham A. D. Bayley H. (2013). A tissue-like printed material. Science 340, 48–52. 10.1126/science.1229495 23559243 PMC3750497

[B40] Wang M. O. Vorwald C. E. Dreher M. L. Mott E. J. Cheng M. H. Cinar A.. (2015). Evaluating 3D-printed biomaterials as scaffolds for vascularized bone tissue engineering. Adv. Mater. Weinheim. 27, 138–144. 10.1002/adma.201403943 25387454 PMC4404492

[B41] Zorlutuna P. Annabi N. Camci-Unal G. Nikkhah M. Cha J. M. Nichol J. W.. (2012). Microfabricated biomaterials for engineering 3D tissues. Adv. Mater. Weinheim. 24, 1782–1804. 10.1002/adma.201104631 22410857 PMC3432416

[B42] Zorlutuna P. Vrana N. E. Khademhosseini A. (2013). The expanding world of tissue engineering: the building blocks and new applications of tissue engineered constructs. IEEE Rev. Biomed. Eng. 6, 47–62. 10.1109/RBME.2012.2233468 23268388 PMC3617045

